# Phosphatidylcholine Biosynthesis in Mitis Group Streptococci via Host Metabolite Scavenging

**DOI:** 10.1128/JB.00495-19

**Published:** 2019-10-21

**Authors:** Luke R. Joyce, Ziqiang Guan, Kelli L. Palmer

**Affiliations:** a Department of Biological Sciences, The University of Texas at Dallas, Richardson, Texas, USA; b Department of Biochemistry, Duke University Medical Center, Durham, North Carolina, USA; University of Illinois at Chicago

**Keywords:** *Streptococcus pneumoniae*, phosphatidylcholine, phospholipids

## Abstract

The mitis group streptococci include the major human pathogen Streptococcus pneumoniae and the opportunistic pathogens Streptococcus mitis and Streptococcus oralis, which are human oral cavity colonizers and agents of bacteremia and infective endocarditis in immunocompromised patients. Bacterial membrane lipids play crucial roles in microbe-host interactions; for many pathogens, however, the composition of the membrane is poorly understood. In this study, we characterized the lipidomes of selected species of mitis group streptococci and investigated the mechanistic basis for biosynthesis of the phospholipid phosphatidylcholine (PC). PC is a major lipid in eukaryotic cellular membranes, but it is considered to be comparatively rare in bacterial taxa. Using liquid chromatography-mass spectrometry in conjunction with stable isotope tracing, we determined that mitis group streptococci synthesize PC via a rare host-metabolite-scavenging pathway, the glycerophosphocholine (GPC) pathway, which is largely uncharacterized in bacteria. Our work demonstrates that mitis group streptococci, including S. pneumoniae, remodel their membranes in response to the major human metabolites GPC and lysophosphatidylcholine.

**IMPORTANCE** We lack fundamental information about the composition of the cellular membrane even for the best-studied pathogens of critical significance for human health. The mitis group streptococci are closely linked to humans in health and disease, but their membrane biology is poorly understood. Here, we demonstrate that these streptococci scavenge major human metabolites and use them to synthesize the membrane phospholipid PC. Our work is significant because it identifies a mechanism by which the major human pathogen S. pneumoniae and the primary human oral colonizers S. mitis and S. oralis remodel their membranes in response to host metabolites.

## Abstract (precis)

We lack fundamental information about the composition of the cellular membrane even for the best-studied pathogens of critical significance for human health. The mitis group streptococci are closely linked to humans in health and disease, but their membrane biology is poorly understood. Here, we demonstrate that these streptococci scavenge major human metabolites and use them to synthesize the membrane phospholipid PC. Our work is significant because it identifies a mechanism by which the major human pathogen S. pneumoniae and the primary human oral colonizers S. mitis and S. oralis remodel their membranes in response to host metabolites.

## INTRODUCTION

The mitis group streptococci are Gram-positive bacteria that natively inhabit the human oral cavity, nasopharynx, and gastrointestinal tract (1). They include the species Streptococcus mitis and Streptococcus oralis, which are among the first colonizers of the human oral cavity from birth and facilitate host-microbe-microbe interactions by creating anchors for biofilm formation with other oral microbiota (2, 3). S. mitis and S. oralis are also opportunistic pathogens that cause bacteremia and infective endocarditis (4–7). The mitis group streptococci also include the major human pathogen Streptococcus pneumoniae. S. pneumoniae has >99% 16S rRNA sequence identity (8–10), exchanges capsule biosynthesis and antibiotic resistance genes (11, 12), and shows antibody cross-reactivity (13) with S. mitis and S. oralis.

**FIG 1:**
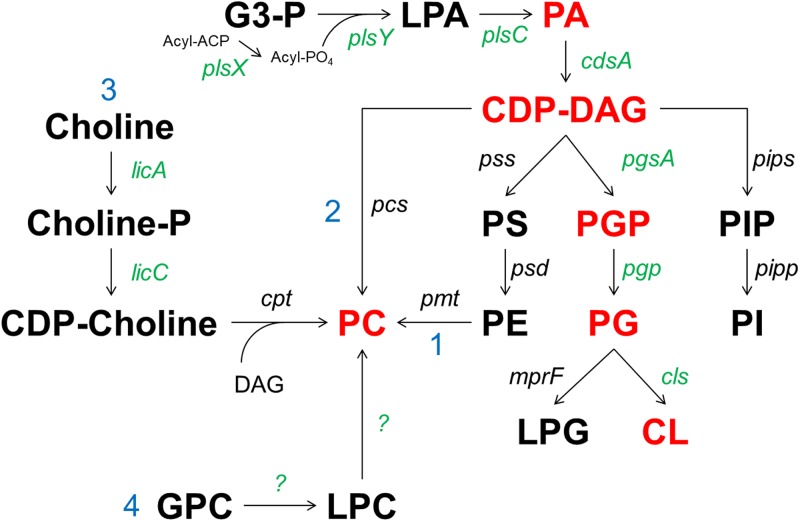
Phospholipid pathways in bacteria, including pathways for PC biosynthesis. Shown are the methylation pathway (pathway 1), the Pcs pathway (pathway 2), the CDP-choline pathway (pathway 3), and the GPC pathway (pathway 4). Lipids detected in THB-cultured SM43 cells are shown in red. Genes present in SM43 are shown in green. PA, phosphatidic acid; PS, phosphatidylserine; G3-P, glycerol-3-phosphate; PGP, PG-3-phosphate; LPG, lysyl-phosphatidylglycerol; PIP, phosphatidylinositol phosphate; PI, phosphatidylinositol; LPC, lysoPC; LPA, lysophosphatidic acid; acyl-ACP, acyl-acyl carrier protein. Other abbreviations are defined in the text.

We, and others, recently reported that certain mitis group streptococci have unusual membrane physiology, in that they can proliferate while lacking the major anionic phospholipids phosphatidylglycerol (PG) and cardiolipin (CL) (14, 15). More specifically, S. mitis and S. oralis can tolerate deletion or inactivation of *cdsA*, the gene encoding phosphatidate cytidylyltransferase (CdsA) (14, 15). CdsA catalyzes synthesis of CDP-diacylglycerol (CDP-DAG), a key intermediate in the synthesis of major phospholipids (Fig. 1). Deletion or inactivation of *cdsA* in mitis group streptococci and the corresponding loss of PG and CL confer high-level resistance (MICs of >256 μg/ml) to daptomycin, a last-line lipopeptide antibiotic (14–16).

Unexpectedly, lipidomic analysis of S. mitis and S. oralis by normal-phase liquid chromatography (NPLC)-electrospray ionization (ESI)-mass spectrometry (MS) revealed the presence of phosphatidylcholine (PC) in wild-type strains and in *cdsA*-null mutants (14). To our knowledge, PC in streptococci had not been described previously. Overall, the lipid compositions of streptococci are understudied and poorly characterized. Previous studies analyzing lipids of streptococci primarily used thin-layer chromatography, whose limitations in analytical sensitivity and molecular specificity prohibit comprehensive lipidomic identification; those studies did not detect PC (17–22). PC is a biologically significant lipid. As a zwitterionic phospholipid, PC promotes bilayer formation (23), reduces the rate of protein folding to allow correct protein configurations (24, 25), aids in resistance to antimicrobials targeting prokaryotic membranes (26), aids in survival of environmental fluctuations such as temperature shifts (27), and is a major component of eukaryotic membranes. There is evidence that PC plays important roles in host-microbe interactions. *Legionella* strains lacking functional PC biosynthesis exhibit decreased virulence because of poor recognition by host macrophages and reduced motility (28). Brucella abortus and Agrobacterium tumefaciens also exhibit diminished virulence when PC biosynthesis is inactivated (29–31). In contrast, Pseudomonas aeruginosa strains lacking PC show no detectable alterations in virulence (32).

Because PC may affect how mitis group streptococci interact with the human host, in this study we investigated the mechanism for PC biosynthesis in these organisms. There are four experimentally confirmed PC biosynthesis pathways in bacteria (Fig. 1), two of which are widespread and well characterized, namely, the phosphatidylethanolamine (PE) methylation pathway (23, 33) and the PC synthase (Pcs) pathway (34, 35). The PE methylation pathway uses PE as a starting substrate; it is methylated via phospholipid *N*-methyltransferase (Pmt) in three subsequent steps to form monomethylphosphatidylethanolamine, dimethylphosphatidylethanolamine, and finally PC, using *S*-adenosylmethionine as the methyl group donor (33). The PE methylation pathway is utilized by mammalian liver cells (23, 36) and bacteria, including Rhodobacter sphaeroides and Sinorhizobium meliloti (23). The Pcs pathway is exclusive to prokaryotes and is a one-step reaction in which a Pcs enzyme condenses choline with CDP-DAG to form PC (34). The presence of either *pmt* or *pcs* genes has been used to identify bacterial taxa likely to produce PC (23).

A third pathway, the CDP-choline pathway (referred to as the Kennedy pathway in eukaryotes), was recently identified in the Gram-negative human oral colonizer Treponema denticola (37). In this pathway, choline is scavenged from the environment and activated to CDP-choline via the LicAC enzymes. Many host-associated bacteria possess LicAC and utilize host-derived choline to decorate a wide range of extracellular structures (23), including the type IV lipoteichoic acid (LTA) of S. pneumoniae, S. mitis, and S. oralis (38–42). In the CDP-choline pathway, CDP-choline is condensed with DAG by a 1,2-DAG cholinephosphotransferase (CPT) to form PC.

A fourth pathway, the glycerophosphocholine (GPC) pathway, has been reported for only two organisms, namely, the Gram-negative plant pathogen Xanthomonas campestris (43) and Saccharomyces cerevisiae (44). In eukaryotic cells, GPC is a breakdown product of choline-containing membrane phospholipids. Yeast can utilize GPC as the source for glycerol-3-phosphate, choline, or phosphate, depending on the environmental conditions (45). GPC is a major human metabolite present in saliva and blood (46, 47). In the GPC pathway, GPC is scavenged from the environment and acylated twice to form the intermediate lysophosphatidylcholine (lysoPC) and then PC. The genetics underlying the GPC pathway in X. campestris have not been fully elucidated. Moser et al. identified two X. campestris acyltransferases that performed the second acylation from lysoPC to PC (43). Yeasts possess a fully elucidated GPC pathway (44, 48).

Here, we use NPLC-ESI-MS and other biochemical and genetic approaches to investigate PC biosynthesis in mitis group streptococci, using type strains and an infective endocarditis isolate (Table 1). We determined that these organisms synthesize PC by the rare GPC pathway via scavenging of the host metabolites GPC and lysoPC.

**TABLE 1:** Summary of major glycolipids and phospholipids detected in mitis group streptococci in routine laboratory media

Streptococcal species	Strain	Source	Growth medium^*a*^	Detection of^*b*^:								
				DAG	MHDAG	DHDAG	PA	PG	CL	C_55_-P	PC	L-PG
S. mitis	ATCC 49456	Type strain	THB	+	+	+	+	+	+	+	+	−
S. oralis	ATCC 35037	Type strain	THB	+	+	+	+	+	+	+	+	−
	1647^*c*^	Endocarditis	THB	+	+	+	+	+	+	+	+	−
	1648^*c*^	Endocarditis	THB	+	+	+	+	+	+	+	+	−
S. pneumoniae	D39	Historical strain	THB+Y	+	+	+	+	+	+	+	+	−
	TIGR4	Bacteremia	THB+Y	+	+	+	+	+	+	+	+	−
Mitis group	1643 (SM43)^*c*^	Endocarditis	THB	+	+	+	+	+	+	+	+	−
	SM43Δ*cdsA*	This study	THB	+	+	+	+	−	−	+	+	−
	SM43Δ*pgsA*	This study	THB	+	+	+	+	−	−	+	+	−

a THB+Y, THB supplemented with 0.5% yeast extract.

b MHDAG, monohexosyldiacylglycerol; DHDAG, dihexosyldiacylglycerol; PA, phosphatidic acid; C_55_-P, undecaprenyl phosphate; L-PG, lysylphosphatidylglycerol; +, detected; −, not detected.

c Lipid profiles were previously reported by Adams et al. (14).

## RESULTS

### PC biosynthesis by model S. mitis and S. oralis strains used in this study.

We previously reported lipidomic analysis by NPLC-ESI-MS of three mitis group infective endocarditis isolates cultured in the rich, undefined, laboratory growth medium Todd-Hewitt broth (THB). We determined that these organisms possessed PC in their membranes (14) (Table 1). We confirmed these results using the S. mitis and S. oralis type strains ATCC 49456 and ATCC 35037, respectively (Table 1).

In this study, we used one of the infective endocarditis isolates, referred to as SM43, for most of our mechanistic studies of PC biosynthesis. Phylogenetic assignments within the mitis group are difficult, due to variable phenotypes and highly conserved 16S rRNA sequences (8, 9). SM43 was initially assigned to the S. mitis species using standard biochemical techniques (49). We analyzed a complete SM43 genome sequence using an average nucleotide identity (ANI) calculator (50, 51). ANI values are used for molecular species definitions (52). Two bacterial strains with ANI of ≥95% in their shared genes are considered to be the same species, and those with ANI of >70% are considered to be the same genus (53, 54). SM43 possesses 94.3% ANI with S. oralis ATCC 35037 and 86.9% ANI with S. mitis ATCC 49456. Based on these data showing a close phylogenetic relationship between SM43 and S. oralis, we refer to SM43 as a mitis group *Streptococcus* strain in this study (Table 1).

### PC biosynthesis in SM43 is not via the PE methylation or Pcs pathway.

The well-characterized PE methylation pathway for PC biosynthesis is catalyzed by the enzyme Pmt (Fig. 1). This pathway is excluded because SM43 does not synthesize PE (14) and does not possess *pmt*.

**FIG 2:**
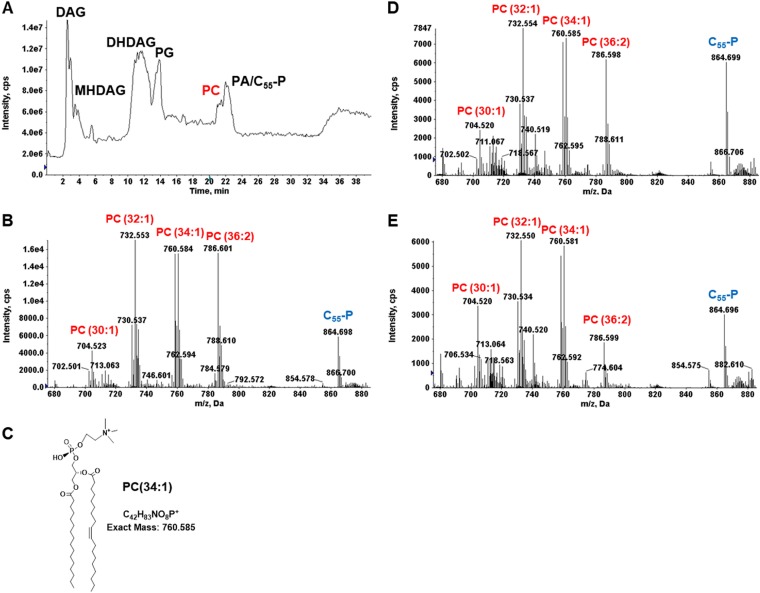
PC species detected in SM43, SM43Δ*cdsA*, and SM43Δ*pgsA*. (A) Positive-mode TIC of SM43 lipids. (B) ESI-MS of PC species in SM43. (C) Chemical structure of PC(34:1). (D) ESI-MS of PC species in SM43Δ*cdsA*. (E) ESI-MS of PC species in SM43Δ*pgsA*. The mass spectra shown were averaged from spectra acquired by NPLC-ESI-MS during the window of 20 to 21 min. PC species were detected by positive-ion ESI-MS as the M^+^ ions. MHDAG, monohexosyldiacylglycerol; DHDAG, dihexosyldiacylglycerol; C_55_-P, undecaprenyl phosphate.

The Pcs pathway requires CDP-DAG and choline as substrates (Fig. 1). Loss-of-function *cdsA* mutants arising spontaneously among mitis group streptococci under daptomycin selection do not synthesize CDP-DAG but still synthesize PC (14). These results indicate that PC biosynthesis in mitis group streptococci is via a CDP-DAG-independent pathway. To confirm these results, we generated a *cdsA* deletion in SM43. PC was detected in both wild-type and Δ*cdsA* SM43 strains cultured in THB. Figure 2A shows the relative abundance of PC in the SM43 membrane in mid-exponential phase. The ESI-MS spectra of the major PC species, including PC(30:1) at *m/z* 704, PC(32:1) at *m/z* 732, PC(34:1) at *m/z* 760, and PC(36:2) at *m/z* 786, are shown for SM43 (Fig. 2B) and S. mitis type strain ATCC 49456 (see Fig. S1A in the supplemental material). The chemical structure of PC(34:1) shown in Fig. 2C was supported by tandem mass spectrometry (MS/MS) (Fig. S1B). The ESI-MS spectrum of PC in SM43Δ*cdsA* is shown in Fig. 2D. Based on these results and the absence of a *pcs* ortholog in the SM43 and ATCC 49456 genomes (Fig. 1), the Pcs pathway is excluded.

### SM43 expresses a partial CDP-choline pathway.

The CDP-choline pathway requires activation of choline to CDP-choline by the LicA and LicC enzymes. This is followed by condensation of CDP-choline with DAG by a 1,2-DAG CPT enzyme to form PC (Fig. 1). S. mitis and S. oralis express *licABC* for the activation of exogenous choline to CDP-choline, which is required for choline decoration of type IV LTA in these organisms and S. pneumoniae (38–42). Given that SM43 expresses *licABC*, we assessed the possibility that the CDP-choline pathway is used for PC biosynthesis in this organism.

The CPT of T. denticola possesses a CDP-alcohol phosphatidyltransferase domain (NCBI superfamily accession no. cl00453) (37). Only one SM43 predicted protein, phosphatidylglycerophosphate synthase (PgsA), possesses this domain. PgsA catalyzes the addition of glycerol phosphate to CDP-DAG to form phosphatidylglycerophosphate (55), which is required for subsequent PG and CL synthesis (Fig. 1).

To investigate whether PgsA has CPT activity in SM43, a 284-bp region of *pgsA* encoding the catalytic domain was replaced with an erythromycin resistance cassette. The *pgsA* mutant has a significant growth defect, with a doubling time almost twice that of the wild-type strain (Fig. S2A). The defect is likely due to PgsA interaction with RodZ in membrane homeostasis, as has been reported for Bacillus subtilis (56). Similar to SM43Δ*cdsA*, PC was present in SM43Δ*pgsA* cultured in THB (Fig. 2E). As expected, the SM43Δ*pgsA* mutant lacked PG and CL (Table 1) and had high-level daptomycin resistance (MIC of >256 μg/ml), in agreement with experiments by Tran et al. utilizing S. mitis/S. oralis strains (57). We conclude that the sole candidate for CPT activity in SM43, PgsA, does not catalyze PC biosynthesis.

**FIG 3:**
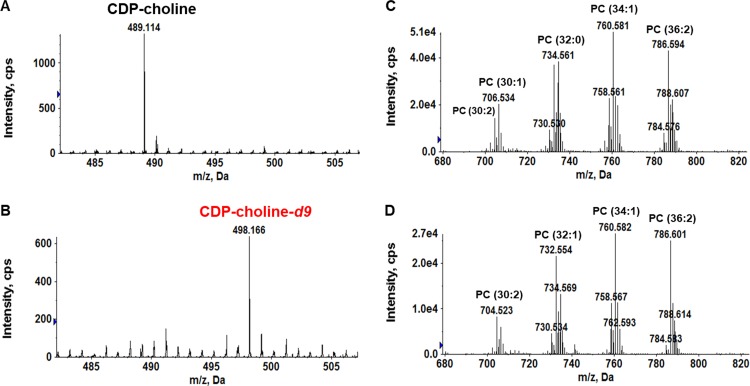
Exogenous deuterated choline (choline-*d*_9_) is used by SM43 to synthesize CDP-choline but not PC. CDP-choline and PC were detected in the soluble-metabolite extract and the lipid extract, respectively. Soluble metabolites were analyzed by RPLC-ESI-MS in positive-ion mode. Lipids were analyzed by NPLC-ESI-MS in positive-ion mode. (A) CDP-choline (M^+^ ion at *m/z* 489.1) present in SM43 cultured in THB. (B) CDP-choline-*d*_9_ (M^+^ ion at *m/z* 498.1) present in SM43 cultured in THB supplemented with 2 mM choline-*d*_9_. Note the expected mass shift (9 Da) between CDP-choline and CDP-choline-*d*_9_. (C) PC species detected in SM43 cultured in THB. (D) PC species detected in SM43 cultured in THB supplemented with choline-*d*_9_. No corresponding mass shift (9 Da) was detected in the PC species, excluding the possibility of SM43 using the CDP-choline pathway for PC synthesis.

To conclusively exclude the CDP-choline pathway, we performed stable isotope labeling experiments. SM43 was cultured in THB supplemented with 2 mM deuterated choline (choline-*d*_9_), in which all nine hydrogen atoms (1 Da) on the three methyl groups are replaced with deuterium atoms (2 Da), thereby increasing the mass of choline by 9 Da. If SM43 utilizes the CDP-choline pathway for PC biosynthesis, then a *m/z* shift of 9 Da would be observed for CDP-choline and PC species in choline-*d*_9_-supplemented SM43 cultures. We observed choline-*d*_9_ incorporation into CDP-choline via a shift of its M^+^ ion from *m/z* 489 (Fig. 3A, no choline-*d*_9_ supplementation) to *m/z* 498 (Fig. 3B, with choline-*d*_9_ supplementation). The identification of CDP-choline and CDP-choline-*d*_9_ was confirmed by MS/MS (Fig. S3). In contrast, no *m/z* shift was observed for PC species between control (Fig. 3C) and choline-*d*_9_-supplemented (Fig. 3D) cultures. These data definitively eliminate the CDP-choline pathway as the SM43 PC biosynthesis pathway.

### SM43 utilizes the GPC-scavenging pathway for PC biosynthesis.

GPC is a major human metabolite that is present in blood and saliva at concentrations of up to 40 μM and 10 μM, respectively (46). Using reverse-phase liquid chromatography (RPLC)-MS, we detected GPC (*m/z* 258.1) in THB (Fig. S4), likely originating from the heart infusion component of the medium. Therefore, GPC is available for scavenging in the medium used for routine SM43 cultures.

**FIG 4:**
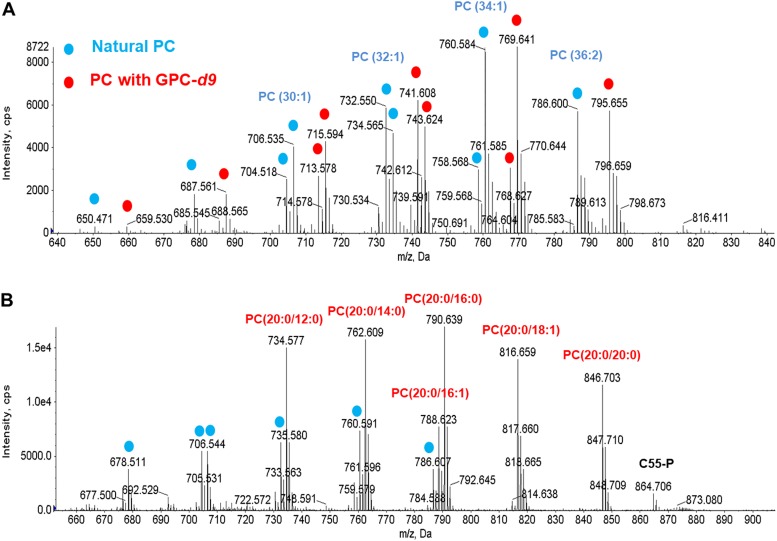
PC species in SM43 cultured in THB supplemented with GPC-*d*_9_ and lysoPC(20:0). (A) ESI-MS detection of PC species in SM43 cultured with GPC-*d*_9_. Blue dots indicate PC species normally detected in SM43 grown in THB, and red dots indicate GPC-*d*_9_-originating PC species. (B) ESI-MS detection of PC species in SM43 cultured in the presence of lysoPC(20:0). Blue dots indicate PC species normally detected in SM43 grown in THB, and red indicates lysoPC(20:0)-originating species. Incorporation of GPC-*d*_9_ and lysoPC(20:0) into PC indicates that the GPC pathway is utilized by SM43 for PC biosynthesis. C_55_-P, undecaprenyl phosphate.

**FIG 5:**
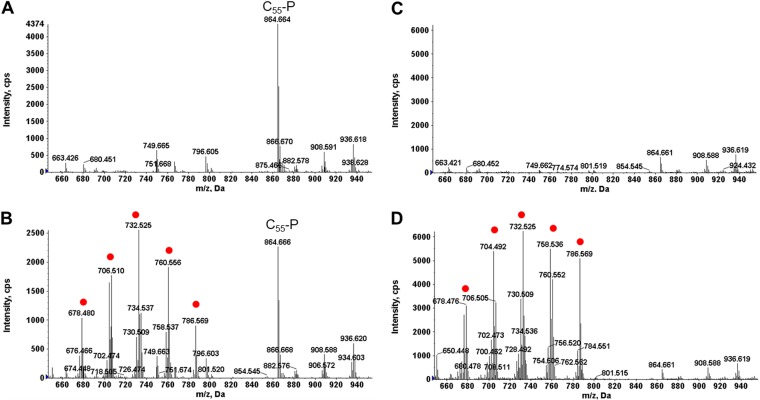
PC species in SM43 and S. mitis ATCC 49456 cultured in DM with or without GPC. PC species (red dots) detected in SM43 (A) and ATCC 49456 (C) cultured in DM and in SM43 (B) and ATCC 49456 (D) cultured in DM supplemented with GPC are shown. PC was detected only when GPC was present in the culture medium. C_55_-P, undecaprenyl phosphate.

To determine whether SM43 utilizes the GPC pathway for PC biosynthesis, we used stable-isotope-labeled GPC to trace the conversion of GPC into PC. SM43 was cultured in THB with and without 0.13 mM GPC-*d*_9_ supplementation. A *m/z* shift of 9 Da was observed for all PC species (Fig. 4A), demonstrating that SM43 uses the GPC pathway for PC biosynthesis. To confirm this result, SM43 and S. mitis ATCC 49456 were cultured in a chemically defined medium containing 0.5 mM choline (58, 59), with or without 0.13 mM GPC supplementation. SM43 and ATCC 49456 synthesized PC only when GPC was present in the defined medium (DM) (Fig. 5 and Table 2). In summary, GPC-*d*_9_ isotope tracking and DM experiments independently confirmed that SM43 utilizes the GPC pathway for PC biosynthesis. Moreover, these results are not strain specific, as the S. mitis type strain also synthesized PC only when GPC was present in the growth environment.

**TABLE 2:** Summary of major glycolipids and phospholipids detected in mitis group streptococci cultured in DM with or without GPC

Streptococcal species	Strain	Source	Growth medium	Detection of^*a*^:								
				DAG	MHDAG	DHDAG	PA	PG	CL	C_55_-P	PC	L-PG
S. mitis	ATCC 49456	Type strain	DM	+	+	+	+	+	+	+	−	−
			DM + GPC	+	+	+	+	+	+	+	+	−
S. oralis	ATCC 35037	Type strain	DM	+	+	+	+	+	+	+	−	−
			DM + GPC	+	+	+	+	+	+	+	+	−
S. pneumoniae	D39	Historical strain	DM	+	+	+	+	+	+	+	−	−
			DM + GPC	+	+	+	+	+	+	+	+	−
Mitis group	1643 (SM43)	Endocarditis	DM	+	+	+	+	+	+	+	−	−
			DM + GPC	+	+	+	+	+	+	+	+	−

a MHDAG, monohexosyldiacylglycerol; DHDAG, dihexosyldiacylglycerol; PA, phosphatidic acid; C_55_-P, undecaprenyl phosphate; L-PG, lysyl-phosphatidylglycerol; +, detected; −, not detected.

PC is not an essential component in the lipid membrane for SM43 and ATCC 49456, as evidenced by their abilities to grow in DM lacking GPC. To assess the impact of PC on growth dynamics, SM43 and ATCC 49456 were cultured in THB and in DM with or without GPC supplementation. GPC presence or absence had no impact on growth (Fig. S2B and C).

**FIG 6:**
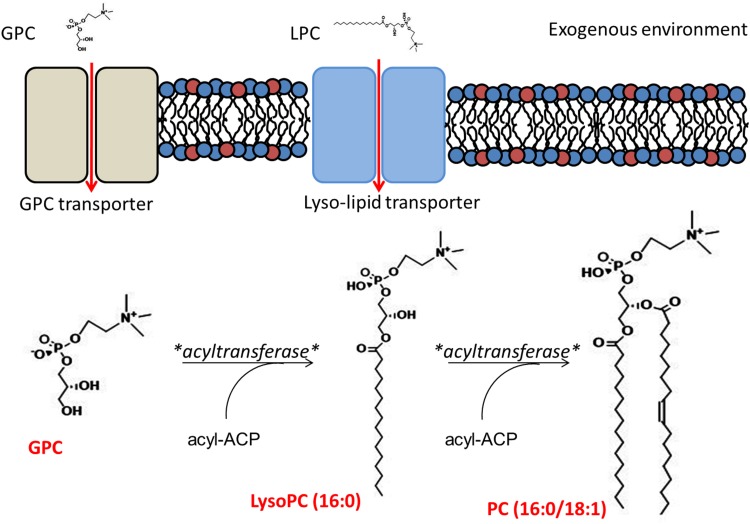
Proposed model for the GPC pathway in mitis group streptococci. Exogenous GPC and lysoPC (LPC) are transported into the cell via unidentified transporters. GPC is sequentially acylated to form lysoPC and PC. Acyl chain lengths vary in the lipid membrane; representative chain lengths are shown.

Since lysoPC is an intermediate in the GPC pathway, we hypothesized that SM43 could also scavenge lysoPC from its environment. LysoPC is present in human blood at up to 200 μM (60). We supplemented THB with lysoPC(20:0), which has an acyl chain that is not usually observed in bacterial membranes. SM43 readily scavenged exogenous lysoPC(20:0) and acylated it to form PC (Fig. 4B). The PG species in the SM43 membrane remained unchanged in the presence of lysoPC(20:0), indicating low transacylation activity (Fig. S5). We conclude that SM43 scavenges both GPC and lysoPC from the environment to synthesize PC (Fig. 6).

### GPC-dependent PC synthesis by S. pneumoniae *and* S. oralis.

**FIG 7:**
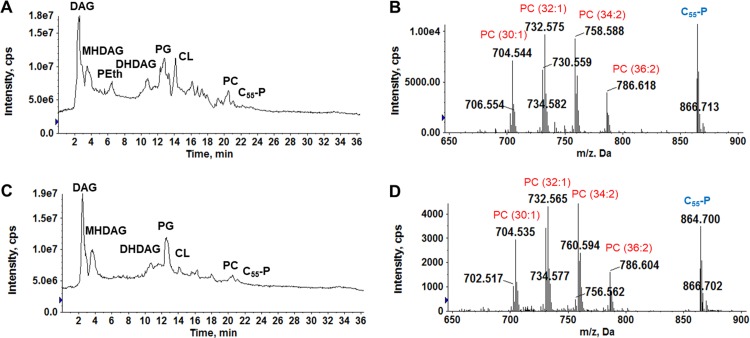
Positive-ion TIC and PC mass spectra for S. pneumoniae D39 and TIGR4 strains in early stationary phase. (A) TIC of S. pneumoniae D39 lipids. (B) ESI-MS of major PC species in S. pneumoniae D39. (C) TIC of S. pneumoniae TIGR4 lipids. (D) ESI-MS of major PC species in S. pneumoniae TIGR4. The mass spectra shown were averaged from spectra acquired by NPLC-ESI-MS during the window of 19.5 to 21 min. PC species were detected by positive-ion ESI-MS as the M^+^ ions, while the coeluting undecaprenyl phosphate (C_55_-P) was detected as the [M+NH_4_]^+^ ion (*m/z* 864.7). MHDAG, monohexosyldiacylglycerol; DHDAG, dihexosyldiacylglycerol; PEth, phosphatidylethanol.

S. pneumoniae is a major human pathogen and a close relative of S. mitis. Surprisingly, there are only a few reports on lipid analysis of S. pneumoniae (17–19), for which thin-layer chromatography was used as the analytical technique. We applied liquid chromatography (LC)-ESI-MS, which has much higher sensitivity and specificity, to investigate the lipidome of S. pneumoniae. PC was present in the membranes of S. pneumoniae D39 (Fig. 7A and B) and TIGR4 (Fig. 7C and D) cultured in THB supplemented with yeast extract (Table 1). Figure 7A and C show the positive-ion total ion chromatogram (TIC) and the relative abundance of PC in D39 and TIGR4, respectively, in early stationary phase. The ESI-MS spectra of the major PC species, including PC(30:1) at *m/z* 704, PC(32:1) at *m/z* 732, PC(34:2) at *m/z* 758, and PC(36:2) at *m/z* 786, are shown for D39 (Fig. 7B) and TIGR4 (Fig. 7D). We identified the presence of PC in infective endocarditis S. oralis isolates 1647 and 1648 in a previous study (14).

**FIG 8:**
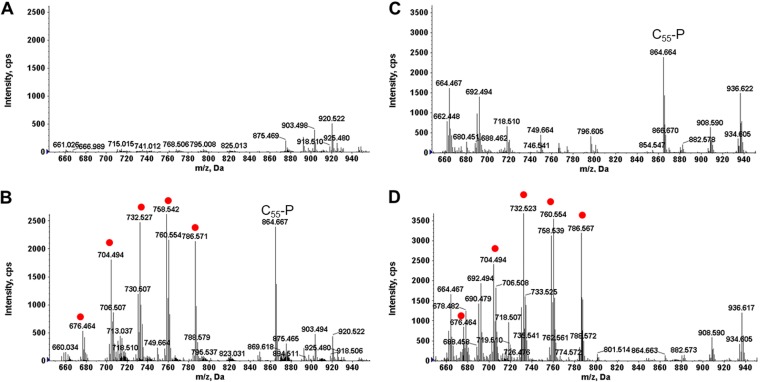
GPC pathway confirmation in S. pneumoniae D39 and S. oralis ATCC 35037. PC species (red dots) detected in D39 (A) and ATCC 35037 (C) cultured in DM and in D39 (B) and ATCC 35037 (D) cultured in DM supplemented with GPC are shown. PC was detected only when GPC was present in the culture medium. C_55_-P, undecaprenyl phosphate.

To determine whether S. pneumoniae and S. oralis utilize the GPC pathway for PC biosynthesis, S. pneumoniae D39 and S. oralis ATCC 35037 were cultured in DM with or without GPC supplementation. When GPC was not available, S. pneumoniae and S. oralis did not synthesize PC (Fig. 8A and C); PC was present only when GPC was available in the medium (Fig. 8B and D). We conclude that S. pneumoniae and S. oralis also scavenge GPC to synthesize PC.

## DISCUSSION

The lipid membrane is a dynamic site of interaction between microbial pathogens and their hosts. For many pathogens, however, the composition of the membrane is poorly understood. In this study, we characterized the lipidomes of selected species of mitis group streptococci and investigated the mechanistic basis for biosynthesis of the phospholipid PC. We found that mitis group streptococci remodel their membrane lipid compositions in response to the host metabolites GPC and lysoPC. To our knowledge, this is the first description of PC in S. pneumoniae, a major human pathogen that has been studied for over a century but whose membrane lipid composition remains poorly understood. There have been very few lipidomic studies performed in S. pneumoniae (17–19), and little is known about how S. pneumoniae remodels its membrane in response to changing environments inside and outside the host. Here, we reported the first identification of PC in S. pneumoniae and demonstrated that S. pneumoniae synthesizes PC only when GPC, a major human metabolite, is available for scavenging.

Very little is known about the GPC pathway in bacteria; however, a complete GPC pathway has been characterized in yeast (44, 48). The pathway includes a dual-substrate transporter, Git1, for the uptake of glycerophosphoinositol and GPC, a GPC acyltransferase referred to as Gpc1, and the acyltransferase Ale1, performing lysoPC acylation. To fully elucidate the GPC pathway in streptococci, identification of transporters for GPC and lysoPC, as well as the acyltransferases, is required. However, no orthologs of the yeast GPC pathway components were identified in the SM43 genome. There are two acyltransferases encoded in the S. mitis genome, PlsY and PlsC, which are responsible for phosphatidic acid biosynthesis in other organisms and may play a role in the acylation of either GPC or lysoPC. In Mycobacterium tuberculosis, a sugar-binding ABC transporter, UgpABCE, transports GPC (61). The UgpB substrate-binding domain is flexible in substrate affinity, binding to phosphate-containing substrates such as *sn*-glycerol 3-phopshate, glycerol 2-phosphate, and GPC (61–63). Given the reduced size of streptococcal genomes, it is possible that GPC uptake is also performed by a transport system with flexible substrate specificity.

Is PC biosynthesis important for mitis group streptococcal virulence? We expect that PC levels in the membrane would affect membrane charge, for example, which could in turn affect biofilm formation and interactions with the host immune system. Moreover, GPC and lysoPC levels vary at different sites within the human body and in health and disease (≤500 μM and ≤200 μM, respectively [46, 60]), which could affect the relative ratios of the zwitterionic PC to the anionic phospholipids PG and CL in mitis group streptococci colonizing these sites. Due to our limited understanding of the genes underlying the GPC pathway, at present our only method to control this pathway is by altering the *in vitro* growth medium. For this reason, *in vivo* studies are not currently feasible. However, by culturing mitis group streptococci in DM with or without GPC, in future studies we can assess the biophysical impact of PC on streptococcal membranes in terms of lipid/microdomain organization, charge, rigidity, and protein composition, which would be informative from a basic science perspective.

Overall, our work highlights the importance of utilizing laboratory culture media that mimic the *in vivo* nutritional environments in which pathogens are found. Our identification of PC in the membranes of mitis group streptococci, including the major human pathogen S. pneumoniae, and their utilization of the rare GPC pathway justify further investigation into streptococcal membrane biology, about which little is known.

## MATERIALS AND METHODS

### Bacterial strains, media, and growth conditions.

The strains and plasmids used in this study are shown in Table S1 in the supplemental material. Streptococcal strains were grown in THB at 37°C in 5% CO_2_ unless otherwise stated. S. pneumoniae THB cultures were supplemented with 0.5% yeast extract. Streptococcal chemically defined medium (58) was diluted from stock as described (59) and supplemented with 0.5 mM choline (referred to as DM), slightly modified from reference 64, unless otherwise stated. Media were supplemented with 130 μM GPC (Sigma-Aldrich) where stated. Erythromycin was used at 20 μg/ml for SM43. Daptomycin susceptibilities were assessed using daptomycin Etest strips (bioMérieux) on Mueller-Hinton agar plates, according to CLSI standards (65).

### Genome sequencing and assembly.

Genomic DNA was isolated using the Qiagen DNeasy blood and tissue kit according to the manufacturer’s protocol, with the exception that cells were pretreated with 180 μl of 50 mg/ml lysozyme, 25 μl of 2,500 U/ml mutanolysin, and 15 μl of 20 mg/ml preboiled RNase A and incubated for 2 h at 37°C. Pacific Biosciences single-molecule real-time (SMRT) sequencing was performed by the Johns Hopkins Genome Core. The SM43 whole genome was assembled using the Unicyler assembly pipeline (66), combining SMRT long reads generated in this study and Illumina reads we had previously generated for SM43 (GenBank accession no. PRJNA354070) (14). Sequencing of SM43Δ*pgsA* was performed by Molecular Research DNA (Shallowater, TX), using Illumina HiSeq paired-end reads (2 by 150 bp).

### Gene deletions in SM43.

Primers used in this study are shown in Table S2. The SM43 *cdsA* deletion construct was designed essentially as described previously (67, 68) (see Text S1 in the supplemental material). For *pgsA* deletion, the protocol was the same as for *cdsA* deletion except that an erythromycin resistance marker with its native promoter was amplified from pG^+^host4 (69) and inserted between the homologous flanking arms, to allow for selection-based screening of putative *pgsA* mutants. In the resulting construct, 284 bp encoding the catalytically active site of PgsA were deleted and replaced with the erythromycin resistance marker. The replacement of *pgsA* was confirmed by whole-genome sequencing.

### Natural transformation.

The natural transformation protocol was as described previously (68), with minor modifications. Briefly, 100 μl of exponential-phase preculture (optical density at 600 nm [OD_600_] of ∼0.5) in THB was frozen with an equal volume of 10% glycerol. Precultures were thawed at room temperature, diluted in 900 μl of THB, further diluted 1:50 in prewarmed 5 ml THB, and incubated for 45 min at 37°C; 500 μl of culture was aliquoted with 1 μl of 1 mg/ml competence-stimulating peptide (DWRISETIRNLIFPRRK) and 1 μg/ml linear DNA construct. Transformation reaction mixtures were cultured for 2 h at 37°C in microcentrifuge tubes before being plated on THB agar, with selection as appropriate.

### Lipidomics.

Unless otherwise noted, lipidomics analyses were performed on overnight cultures in stationary phase. Centrifugation was performed using a Sorvall RC6+ centrifuge. Cultures were pelleted at 4,280 × *g* for 5 min at room temperature. The supernatants were removed and stored at –80°C until acidic Bligh-Dyer lipid extractions were performed as described previously (70), with minor modifications (see Text S1).

NPLC was performed on an Agilent 1200 quaternary LC system equipped with an Ascentis silica high-performance liquid chromatography (HPLC) column (5 μm, 25 cm by 2.1 mm; Sigma-Aldrich), as described previously (70, 71). Data analysis was performed using Analyst TF1.5 software (Sciex, Framingham, MA) (see Text S1).

### Metabolite extractions.

Cultures were pelleted at 4,280 × *g* in a Sorvall RC6+ floor centrifuge at room temperature, washed once with 1× phosphate-buffered saline, and transferred to 1.5-ml microcentrifuge tubes. Cells were pelleted and frozen at –80°C until use. Metabolite extraction was performed as described previously (72), with minor modifications (see Text S1).

### RPLC-ESI-MS analysis.

RPLC-ESI-MS analysis of water-soluble metabolites was performed using a Shimadzu LC system (including a solvent degasser, two LC-10A pumps, and a SCL-10A system controller) coupled to a TripleTOF 5600 mass spectrometer (Sciex) (see Text S1 for detailed information on LC flow rate and mass spectrometer settings). Data acquisition and analysis were performed using Analyst TF1.5 software (Sciex).

### Deuterated isotope and lysoPC tracking.

Deuterated isotope tracking was performed by addition of 2 mM choline-*d*_9_ (Sigma-Aldrich) in 50 ml of THB; 3.7 mM GPC-*d*_9_ (Toronto Research Chemicals) was added to 15 ml of THB to yield a final concentration of 130 μM. Cultures were grown in 5% CO_2_ at 37°C for 18 h before 10 ml of choline-*d*_9_ culture or 5 ml of GPC-*d*_9_ culture was removed for metabolite extraction and the remaining culture was pelleted for lipid extraction. LysoPC(20:0) was obtained from Avanti Polar Lipids. Cultures were supplemented with 2 mg of lysoPC per 15 ml of THB, unless otherwise stated, and incubated overnight at 37°C in 5% CO_2_. Lipid extractions were performed as described above.

### Growth curves.

Individual colonies were incubated overnight in 5 ml of DM. Cultures were diluted to a starting OD_600_ of 0.05 in 20 ml of prewarmed DM, DM with 130 μM GPC, or THB. The OD_600_ was monitored every 1 h using a Thermo Scientific Genesys 30 spectrophotometer. Growth curves were performed in biological triplicates for each strain.

### ANI analysis.

The ANI calculator (50, 51) was used with default parameters to analyze the following genomes: SM43, S. oralis ATCC 35037 (GenBank accession no. PRJNA38733), and S. mitis ATCC 49456 (GenBank accession no. PRJNA173).

### Accession number(s).

The SM43 whole-genome sequence generated in this study has been deposited in GenBank under accession no. CP040231. Illumina and SMRT sequence reads generated in this study have been deposited in the Sequence Read Archive under accession no. PRJNA542100.

## Supplementary Material

Supplemental file 1
