# Supplementary material for: Phosphatidylcholine Biosynthesis in Mitis Group Streptococci via Host Metabolite Scavenging
Source: J Bacteriol. 2019 Oct 21;201(22):e00495-19. doi: 10.1128/JB.00495-19 (PMC6805115; doi:10.1128/JB.00495-19)
Supplement: Supplemental file 1 [file JB.00495-19-s0001.pdf]

Supplemental information

Phosphatidylcholine biosynthesis in Mitis group streptococci via host metabolite scavenging

Luke R. Joyce[a], Ziqiang Guan[b]#, and Kelli L. Palmer[a]#

[a]Department of Biological Sciences, The University of Texas at Dallas, Richardson, Texas, USA

[b]Department of Biochemistry, Duke University Medical Center, Durham, NC[2]

Running title: Phosphatidylcholine biosynthesis in streptococci

#Corresponding authors:

Ziqiang Guan: Ziqiang.Guan@duke.edu

Kelli Palmer: Kelli.Palmer@utdallas.edu

Text S1: Supplementary Materials and Methods.

Acidic Bligh-Dyer extractions. Centrifugation was performed using a Sorvall RC6+ centrifuge. Cultures were pelleted at 4,280 x *g* for 5 min at room temperature. The supernatants were removed and stored at -80°C until acidic Bligh-Dyer lipid extractions were performed as described (1) with minor modifications. Cell pellets were resuspended in 1X PBS (Sigma-Aldrich) and transferred to Coring Pyrex glass tubes with PTFR-lined caps (VWR), followed by 1:2 vol:vol chloroform:methanol addition. Single phase extractions were vortexed periodically and incubated at room temperature for 15 minutes before 500 x *g* centrifugation for 10 min. A two-phase Bligh-Dyer was achieved by addition of 100 µl 37% HCL, 1 mL $CHCl_3$, and 900 µl of 1X PBS, which was then vortexed and centrifuged for 5 min at 500 x *g.* The lower phase was removed to a new tube and dried under nitrogen before being stored at -80°C prior to lipidomic analysis.

Normal phase Liquid Chromatography/ Mass Spectrometry. Normal-phase LC/MS was performed on an Agilent 1200 quaternary LC system equipped with an Ascentis silica high-performance liquid chromatography (HPLC) column (5m; 25 cm by 2.1 mm; Sigma-Aldrich) as described previously (1, 2). Briefly, mobile phase A consisted of chloroform-methanol-aqueous ammonium hydroxide (800:195:5, vol/vol), mobile phase B consisted of chloroform-methanol-water-aqueous ammonium hydroxide (600: 340:50:5, vol/vol), and mobile phase C consisted of chloroform-methanol-water-aqueous ammonium hydroxide (450:450:95:5, vol/vol/vol/vol). The elution program

consisted of the following: 100% mobile phase A was held isocratically for 2 min, then linearly increased to 100% mobile phase B over 14 min, and held at 100% mobile phase B for 11 min. The LC gradient was then changed to 100% mobile phase C over 3 min, held at 100% mobile phase C for 3 min, and, finally, returned to 100% mobile phase A over 0.5 min and held at 100% mobile phase A for 5 min. The LC eluent (with a total flow rate of 300 ml/min) was introduced into the ESI source of a high-resolution TripleTOF5600 mass spectrometer (Sciex, Framingham, MA). Instrumental settings for negative-ion ESI and MS/MS analysis of lipid species were as follows: IS = -4,500 V, CUR = 20 psi, GSI = 20 psi, DP = -55 V, and FP = -150V. The MS/MS analysis used nitrogen as the collision gas. Data analysis was performed using Analyst TF1.5 software (Sciex, Framingham, MA).

Metabolite extractions. Briefly, cell pellets were resuspended in 300 µL super-chilled methanol:water (vol:vol 4:1) via vortexing and pipetting, and stored on dry ice for 15 min. Suspensions were centrifuged at 17,136 x *g* at 4°C for 5 min in a table top centrifuge. The supernatant was removed to a pre-chilled Corning Pyrex glass tube and kept on dry ice. A further 2 resuspensions in 200 µL methanol:water (vol:vol 4:1) were performed. Pooled supernatants were dried under nitrogen at room temperature and stored at -80°C prior to analysis.

Reverse phase LC-ESI/MS analysis. RPLC-ESI/MS analysis of water-soluble metabolites was performed using a Shimadzu LC system (comprising a solvent

degasser, two LC-10A pumps and a SCL-10A system controller) coupled to a TripleTOF5600 mass spectrometer (Sciex, Framingham, MA). LC was operated at a flow rate of 200 μl/min with a linear gradient as follows: 100% of mobile phase A was held isocratically for 2 min and then linearly increased to 100% mobile phase B over 14 min and held at 100% B for 4 min. Mobile phase A consisted of methanol/acetonitrile/aqueous 1mM ammonium acetate (60/20/20, v/v/v). Mobile phase B consisted of 100% ethanol containing 1 mM ammonium acetate. A Zorbax SB-C8 reversed-phase column (5 μm, 2.1 x 50 mm) was obtained from Agilent (Palo Alto, CA). The LC eluent was introduced into the ESI source of the mass spectrometer. Instrument settings for negative ion ESI/MS and MS/MS analysis of lipid species were as follows: Ion spray voltage (IS) = -4500 V; Curtain gas (CUR) = 20 psi; Ion source gas 1 (GS1) = 20 psi; De-clustering potential (DP) = -55 V; Focusing potential (FP) = -150 V. Data acquisition and analysis were performed using the Analyst TF1.5 software (Sciex, Framingham, MA).

Deletion of SM43 *cdsA*. Primers used in this study are shown in Table S2. The *cdsA* deletion construct was designed essentially as previously described (3, 4). Approximately 2 Kb flanking regions on either side of the gene were amplified using Phusion polymerase (Thermo Fisher). PCR products were digested with restriction enzymes (New England Biolabs) and ligated using T4 DNA ligase (New England Biolabs). Ligations were amplified by PCR utilizing the 5’ and 3’ most primers with Phusion polymerase to obtain a linear product. The PCR product was analyzed on a 1% agarose gel and extracted using the QIAquick Gel Extraction Kit per the manufacturer's

protocol. The linear construct was transformed into SM43 via natural transformation, described below. Transformation plates were incubated overnight. Putative transformant colonies were inoculated into THB and screened via PCR for the clean deletion of *cdsA*. The *cdsA* regions of putative mutants were sequenced for confirmation (Massachusetts General Hospital DNA Core).

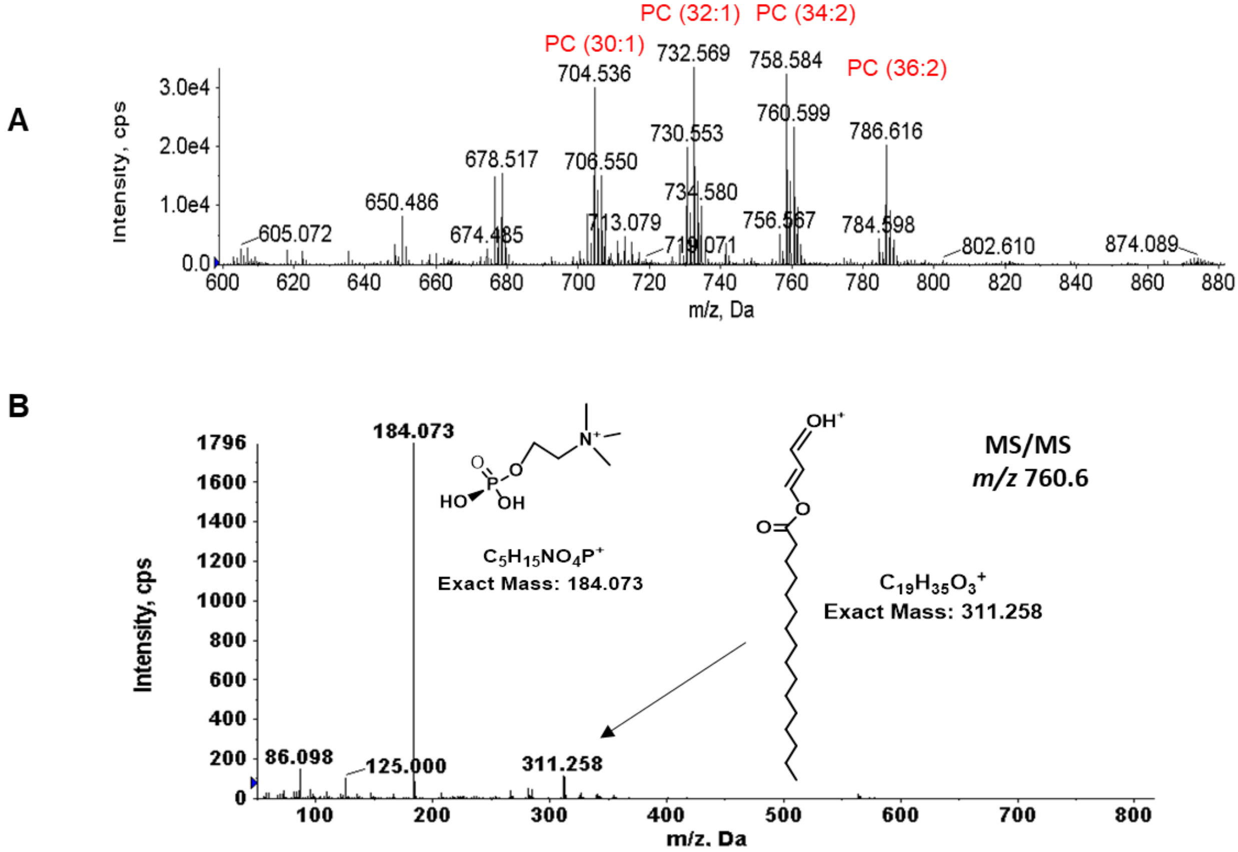

Fig S1. Phosphatidylcholine (PC) species in *S. mitis* ATCC 49456. A) *S. mitis* ATCC 49456, B) MS/MS daughter ion fragmentation of PC (34:1) *m/z* 760.6 in SM43. The mass spectra shown are averaged from spectra acquired by NPLC-ESI/MS during the 20–21 min window. PC species are detected by positive ion ESI/MS as the M^+ ions.

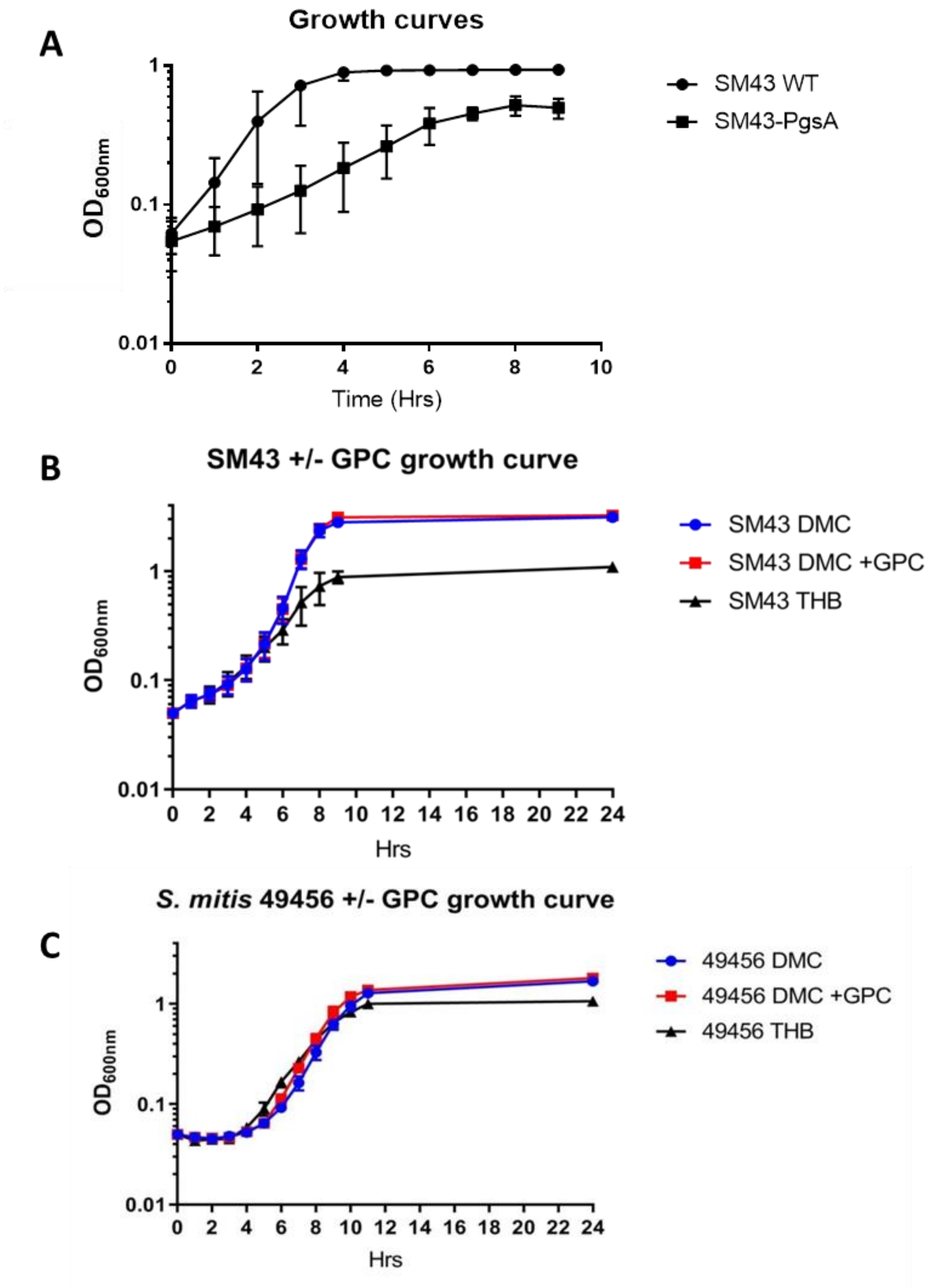

Fig S2. **Growth curves.** A) Growth curves for SM43 and SM43Δ*pgsA* in THB. B) Growth curves of SM43 in THB, and in defined medium (DM) with and without GPC supplementation and C) Growth curves of ATCC49456 in THB, and in defined medium (DM) with and without GPC supplementation. OD_{600nm} readings were taken every hour,

with a starting inoculum of OD_{600nm} 0.05. Growth curves were performed as biological triplicate. Error bars indicate standard deviation.

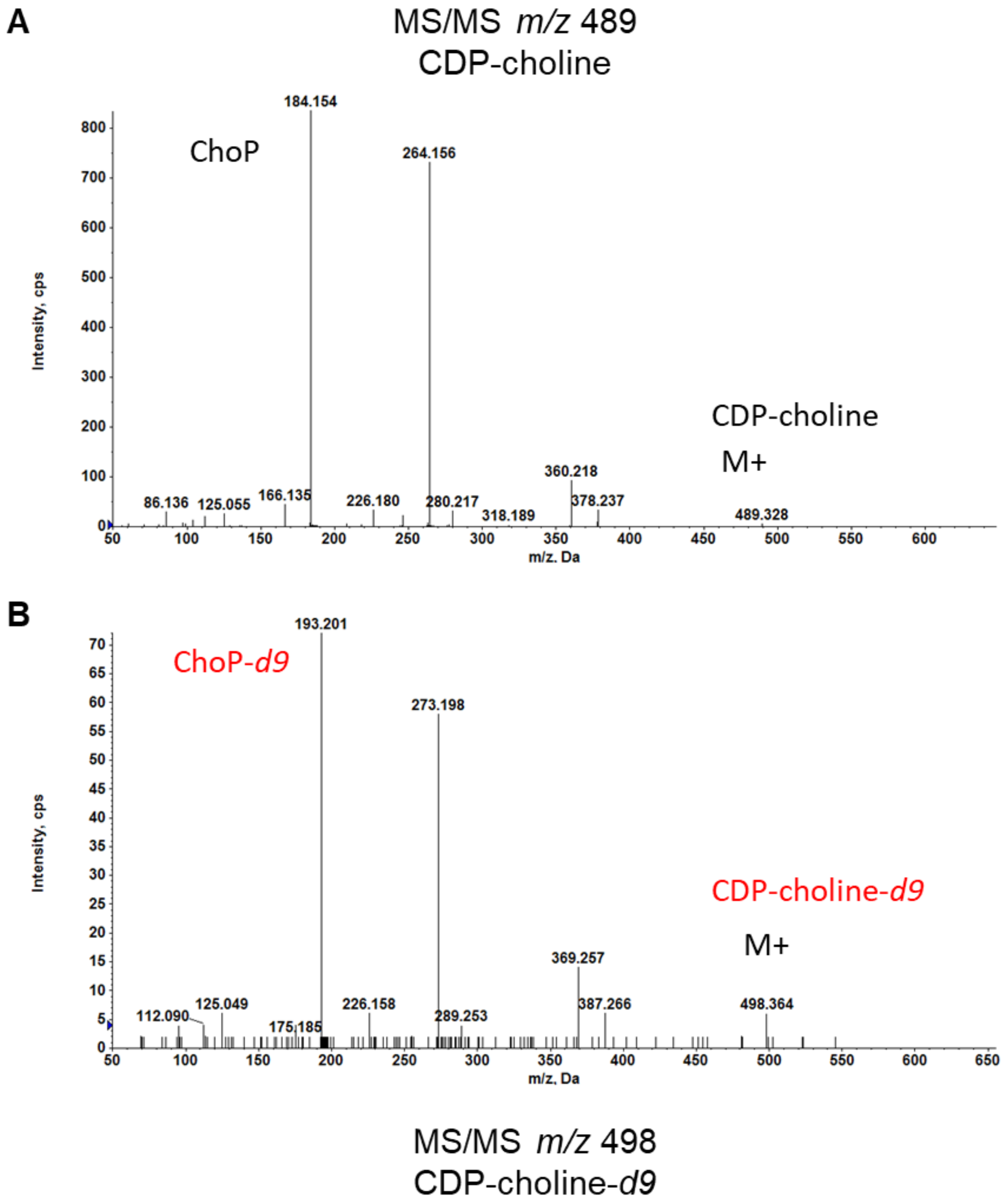

Fig S3. MS/MS fragmentation of CDP-choline and CDP-choline-*d9*. A) MS/MS of CDP-choline (*m/z* 489) B) MS/MS of CDP-choline-*d9* (*m/z* 498). Note the mass shift of 9-*da* for all major MS/MS peaks in B.

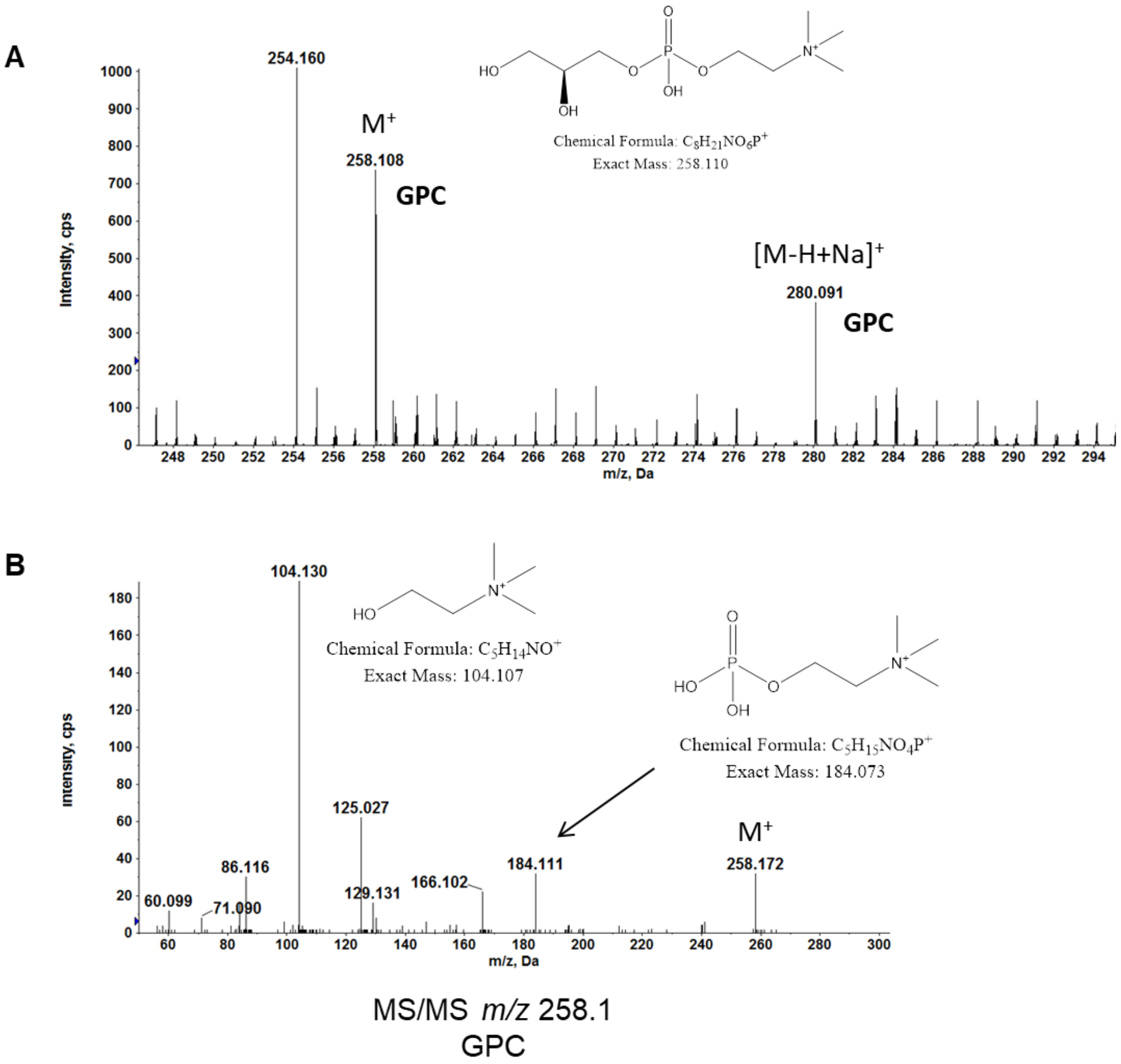

Fig S4. MS detection of GPC in THB. The soluble-metabolite extract of THB was analyzed by reverse phase LC/MS. A) GPC is detected by positive ion ESI/MS as the M^+ ion at *m/z* 258.108 and $[M\text{-}H\text{+}Na]^+$ ion at *m/z* 280.091. B) MS/MS spectrum of the M^+ ion (*m/z* 258.1) of GPC. The chemical structures of major fragments are shown.

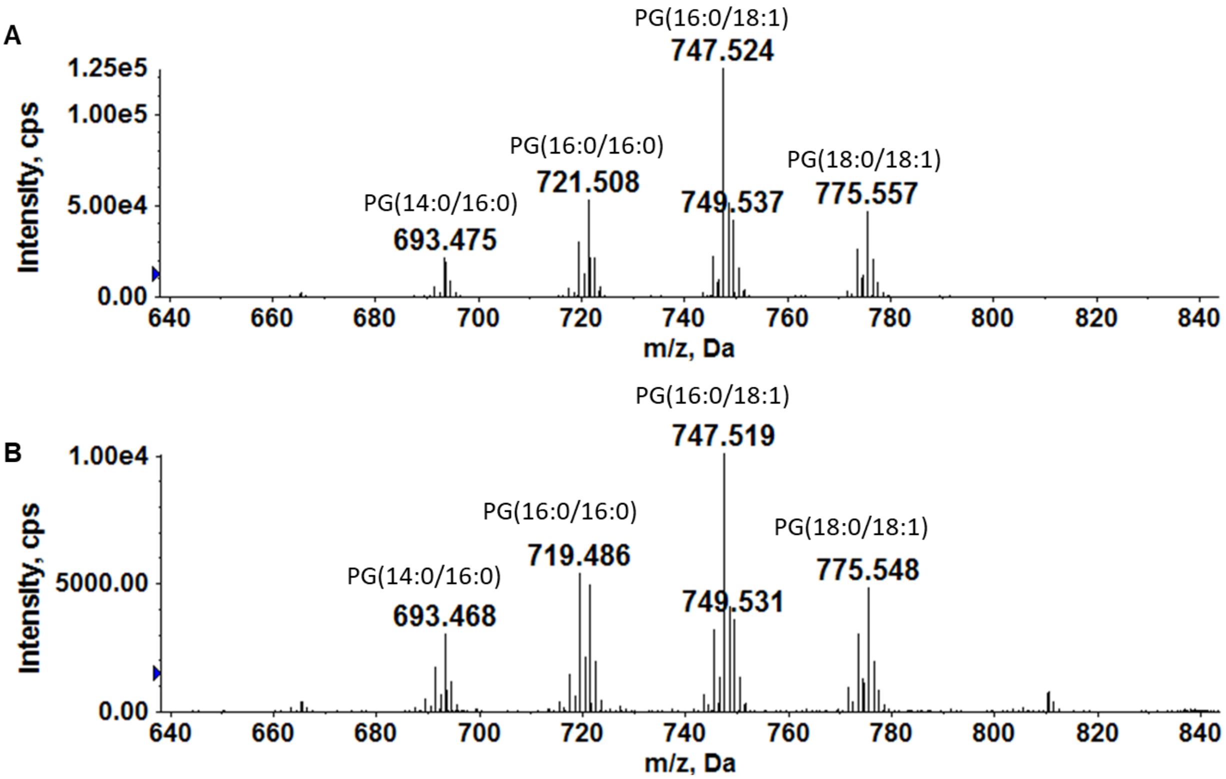

Fig S5. ESI/MS of phosphatidylglycerol (PG) species of SM43 cultured in THB with and without lysoPC (20:0) addition. A) ESI/MS detection of PG species in SM43 cultured in THB. B) ESI/MS detection of PG species in SM43 cultured in THB in the presence of lysoPC (20:0). In sharp contrast to what is observed for PC, there is no appreciable incorporation of the 20:0-acyl chain into PG in the presence of lysoPC (20:0). The lack of incorporation of C20:0 into PG indicates the insignificant transacylation activity of SM43. PG species are detected by negative ion ESI/MS as the $[M-H]^-$ ions.

Table S1. Strains and plasmids used in this study.

**Organism**	**Strain**	**Description**	**Ref**
Mitis group streptococci	1643 (SM43)	Wild-type infective endocarditis isolate	(5)
	1643Δ*cdsA*	*cdsA* deletion strain	This work
	1643Δ*pgsA::ermB*	*pgsA* deletion strain, replaced with *ermB*. Erm^{r}	This work
S. mitis	ATCC 49456 (NCTC 12261)	Wild-type *S. mitis* type strain from ATCC	(6)
S. oralis	ATCC 35037 (NCTC 11427)	Wild-type *S. oralis* type strain from ATCC	(7)
S. pneumoniae	D39	Wild type, historical strain	(8)
	TIGR4	Wild type, bloodstream isolate	(9)
**Plasmid**	**Description**		**Ref**
pG^{+}host 4	Encodes *ermB*		(10)

Table S2. Primers used in this study.

**Primer**	**Sequence (5'-3')**	**Use**
L_Updel_F	TTTACGTGAATATATCGGT	SM43 upstream of *cdsA* region amplification
L_Updel_R PstI	ACGTCACTGCAGATCCTTGGTCATATCTTCTC	SM43 upstream of *cdsA* region amplification
L_Dwndel_F PstI	ACGTCACTGCAGCCAATGATGCACTTATTC	SM43 downstream of *cdsA* region amplification
L_Dwndel_R	CTTGATTTCTTCTTGCAC	SM43 downstream of *cdsA* region amplification
L1_R	CGTACATATCTTCGACTGTC	*cdsA* knockout sequencing with L_Updel_F
L2_F	CCTTCGTACCATGATTGAG	*cdsA* knockout sequencing
L2_R	GTCAGATTTTCCCATTTTTC	*cdsA* knockout sequencing
L3_F	TTTAGAGAATGAGGACCG	*cdsA* knockout sequencing
L3_R	CATATCTTCTGTGATGT	*cdsA* knockout sequencing
L4_F	ATGGTGGTCGTGCTGAA	*cdsA* knockout sequencing
L4_R	GATACTGTACATCCAAAGG	*cdsA* knockout sequencing
L5_F	CAGTAGATCACGATGCAAC	*cdsA* knockout sequencing
L5_R	GTCGCATAATGTCATTCC	*cdsA* knockout sequencing
L6_F	GTGTAGTTATCATGGTTG	*cdsA* knockout sequencing with L_Dwndel_R
L7F	CAAACGGCTCTTCCTATGC	*cdsA* knockout sequencing
L7R	CTGATAACTTGAGCATCG	*cdsA* knockout sequencing
SM43cdsA_reseq_F	TCAGGTCTATATGCATCTG	*cdsA* upstream resequence
SM43cdsA_reseq_R	GCTTCATCAAAATCAGG	*cdsA* upstream resequence
PgsA_updel_F	CTTTACTCCCTAAGCAAG	SM43 upstream of *pgsA* region amplification
PgsA_updel_R_PstI	ACGTCACTGCAGTCTACCAATAGTTAATACAT TAGG	SM43 upstream of *pgsA* region amplification
PgsA_dwndel_F_XmaI	ACGTCACCCGGGTTGTTGCTTGTTGAGACG	SM43 downstream of *pgsA* region amplification
PgsA_dwndel_R	GATCAAGAAGAACATGGA	SM43 downstream of *pgsA* region amplification
Erm_F_XmaI	ACGTCACCCGGGTAACGATCACTCATCATG	*erm* amplification from pG^{+}host4 plasmid
Erm_R_PstI	ACGTCACTGCAGCAAGTTAAGGGATGCAGT	*erm* amplification from pG^{+}host4 plasmid

References

1. Tan BK, Bogdanov M, Zhao J, Dowhan W, Raetz CRH, Guan Z. 2012. Discovery of a cardiolipin synthase utilizing phosphatidylethanolamine and phosphatidylglycerol as substrates. Proc Natl Acad Sci 109:16504–16509.

2. Li C, Tan BK, Zhao J, Guan Z. 2016. In vivo and in vitro synthesis of phosphatidylglycerol by an *Escherichia coli* cardiolipin synthase. J Biol Chem 291:25144–25153.

3. Morrison DA, Khan R, Junges R, Åmdal HA, Petersen FC. 2015. Genome editing by natural genetic transformation in *Streptococcus mutans*. J Microbiol Methods 119:134–141.

4. Salvadori G, Junges R, Morrison DA, Petersen FC. 2016. Overcoming the barrier of low efficiency during genetic transformation of *Streptococcus mitis*. Front Microbiol 7:1009.

5. Akins RL, Katz BD, Monahan C, Alexander D. 2015. Characterization of high-level daptomycin resistance in viridans group streptococci developed upon in vitro exposure to daptomycin. Antimicrob Agents Chemother 59:2102–2112.

6. Kilian M, Mikkelsen L, Henrichsen J. 1989. Taxonomic Study of Viridans Streptococci: Description of *Streptococcus gordonii* sp. nov. and Emended Descriptions of *Streptococcus sanguis* (White and Niven 1946), *Streptococcus oralis* (Bridge and Sneath 1982), and *Streptococcus mitis* (Andrewes and Horder 1906). Int J Syst Bacteriol 39:471–484.

7. Bridge PD, Sneath PH. 1982. *Streptococcus gallinarum* sp. nov. and *Streptococcus oralis* sp. nov. Int J Syst Bacteriol 32:410–415.

8. Lanie JA, Ng WL, Kazmierczak KM, Andrzejewski TM, Davidsen TM, Wayne KJ, Tettelin H, Glass JI, Winkler ME. 2007. Genome sequence of Avery's virulent serotype 2 strain D39 of *Streptococcus pneumoniae* and comparison with that of unencapsulated laboratory strain R6. J Bacteriol 189:38–51.

9. Tettelin H, Nelson KE, Paulsen IT, Eisen JA, Read TD, Peterson S, Heidelberg J, Deboy RT, Haft DH, Dodson RJ, Durkin AS, Gwinn M, Kolonay JF, Nelson WC, Peterson JD, Umayam LA, White O, Salzberg SL, Lewis MR, Radune D, Holtzapple E, Khouri H, Wolf AM, Utterback TR, Hansen CL, Mcdonald LA, Feldblyum T V, Angiuoli S, Dickinson T, Hickey EK, Holt IE, Loftus BJ, Yang F, Smith HO, Venter JC, Dougherty BA, Morrison DA, Hollingshead SK, M. FC. 2001. Complete Genome Sequence of a Virulent Isolate of *Streptococcus pneumoniae*. Science (80-) 293:498–507.

10. Biswas I, Gruss A, Ehrlich SD, Maguin E. 1993. High-efficiency gene inactivation and replacement system for gram-positive bacteria. J Bacteriol 175:3628–3635.
